# Effect of Different Desensitizer Treatments on the Shear Bond Strength of Orthodontic Metal Brackets Following In-Office Bleaching: An *In Vitro* Study

**DOI:** 10.1055/s-0043-1776119

**Published:** 2023-12-04

**Authors:** Septia Emi Ambersari, Dyah Karunia, Ananto Ali Alhasyimi

**Affiliations:** 1 Department of Orthodontics, Faculty of Dentistry, Universitas Gadjah Mada, Yogyakarta, Indonesia

**Keywords:** desensitizing agent, in-office bleaching, shear bond strength

## Abstract

**Objective** One of the adverse effects of in-office bleaching is tooth hypersensitivity, which could be neutralized with a desensitizing agent. In-office bleaching and the application of desensitizing agents will affect the morphology of the enamel surface. These morphological changes have potential effects on the shear bond strength (SBS) and bonding of orthodontic brackets. This study analyzed the effects of fluoride and nonfluoride desensitizing agents after in-office bleaching on the SBS of metal brackets with resin composite cementation.

**Materials and Methods** Twenty-seven postextraction premolars ( *n* = 27) were bleached with 37% hydrogen peroxide and then divided into three groups: control group (group 1), fluoride-based desensitizing agent (group 2), and nonfluoride-based desensitizing agent (group 3). After treatment, the brackets were bonded using Transbond, and then the SBS test was performed using a universal testing machine. The adhesive remnant index (ARI) was analyzed after the SBS test, whereas enamel morphological changes were observed under a scanning electron microscope. After this assessment, energy-dispersive X-ray spectroscopy was conducted to determine calcium and phosphorus elements in the enamel surface after treatments. SBS data were analyzed using the one-way analysis of variance, followed by the Tukey test, whereas ARI scores were subjected to the Kruskal–Wallis test and the Mann–Whitney test with a significance level of 5%.

**Results** The SBS increased significantly in group 2 compared with groups 1 and 3 ( *p* < 0.05). In group 2, an ARI of 2 (55.56%) was frequent, whereas in group 3, an ARI of 3 (55.56%) appeared most frequently.

**Conclusion** The application of the desensitizing agent fluoride after in-office bleaching increased the SBS and more adhesive remains on the tooth surface compared with that when nonfluoride desensitizing agents were applied on metal brackets with composite resin cementation.

## Introduction

At present, orthodontic treatment is one of the most desirable dental procedures worldwide. Having a better facial and dental appearance is the primary motivation for seeking orthodontic treatment. 1 In recent years, community awareness of esthetics has increased. This has resulted in an increased demand for orthodontic treatment. 2 Everyone desires properly aligned, whiter, and brighter teeth, which symbolize vitality, health, and physical beauty. Consequently, some adults who are interested in orthodontic treatment may have had their teeth bleached or may be interested in bleaching. 3 4 In-office bleaching has benefits such as immediate effect and simpler application, which contribute to its higher use than home bleaching. 5 Bleaching agents generate free radicals, such as hydrogen ions and reactive oxygen species, which break double bonds into single bonds that are easily disrupted by chemical oxidation. Large molecules (chromophores) are disassembled into smaller molecules that absorb less light, resulting in less discoloration. However, following bleaching, free radicals and residual peroxides on the enamel surface impede the production of resin tags and the polymerization of resin monomers during the bonding process, resulting in decreased shear bond strength (SBS). 6 7 The bonding of orthodontic brackets to the enamel is the most intricate phase of orthodontic treatment, and the SBS plays an important role. The SBS is one of the main factors to consider in the use of adhesive materials. The attachment of orthodontic brackets must withstand the forces applied during orthodontic treatment. The ideal orthodontic adhesive material should have an adequate bond but must protect the enamel from damage during the bracket debonding process. 8 As a result of decreased SBS, bracket detachment occurs, which disrupts the treatment process, prolongs its duration, and wastes a substantial amount of time. The prevalence of this complication ranges from 0.5 to 17.6%. 9

In recent decades, tooth bleaching has become one of the most successful and widely accepted cosmetic dental treatments; however, bleaching could reduce the wear resistance of the enamel and dentin, increase surface roughness, reduce microhardness, and cause histomorphological changes. 10 Mullins et al showed decreased bracket attachment after bleaching compared with those without bleaching. 11 Decreased bracket attachment is caused by morphological structural changes in the enamel surface. It is caused by the presence of free radical residues in bleaching procedures, which causes loss of prismatic formations, changes in organic components, calcium loss, and decreased microhardness of the enamel. 12 13 Owing to increased enamel and dentin permeability caused by bleaching agents, tooth sensitivity is one of the unfavorable effects of bleaching. 14 Posttreatment sensitivity is typically caused by small microscopic enamel defects and subsurface pores that allow the bleaching agent to penetrate the dental tubules and eventually the pulp, causing reversible pulpitis and subsequent dental thermal sensitivity. 15 This can be preserved through the use of desensitizing agents, mainly potassium nitrate, which reduces sensitivity by inhibiting the ability of the nerve fibers in the dental pulp to deliver pain, and fluorides, which interfere with the dentinal tubules and consequently block pain. 16 17 Kristanti et al showed that pain can be alleviated by using desensitizing compounds such as casein phosphopeptide-amorphous calcium fluoride phosphate (CPP-ACFP) or fluoride-free materials such as CPP-amorphous calcium phosphate (CPP-ACP). 18 The ability of desensitizing agents such as CPP-ACP and CPP-ACFP in overcoming pain causes frequent use of desensitizing agents after bleaching. The application of desensitizing agents such as acidulated phosphate fluoride and CPP-ACP increased the SBS of the bracket compared with the control group. 19

Morphological changes in the enamel and dentin after bleaching followed by the use of desensitizing agents must be a concern in orthodontics. Patients requiring orthodontic treatment place a high value on their appearance. Some of them have discolored teeth and request bleaching before receiving orthodontic treatment because they do not wish to have discolored teeth during their orthodontic treatment, which can last over 3 years. 20 Consequently, evaluating the effect of bleaching treatments in combination with desensitizing compounds on the bond strength of metal brackets is essential. This study examined the effect of fluoride and nonfluoride desensitizing agents on the SBS of metal brackets with resin composite cementation after in-office bleaching.

## Material and Methods

### Sample Preparation

This study is an experimental laboratory research. This study was ethically approved by the Ethics Committee of Research of Dentistry Faculty, Universitas Gadjah Mada, Yogyakarta, Indonesia, on October 7, 2022, with protocol no. 177/KE/FKG-UGM/EH/2022. A total of 27 extracted first upper premolars were collected and divided into three groups. Group 1 (control group) did not use desensitizing agents after bleaching, group 2 used fluoride-based desensitizing agent (CPP-ACFP gel, GC Tooth Mousse Plus; GC Int Corp, Tokyo, Japan) after bleaching, and group 3 used nonfluoride-based desensitizing agent (CPP-ACP gel, GC Tooth Mousse; GC Int Corp) after bleaching. The teeth had normal anatomy and healthy enamel on the buccal side, without any caries, enamel cracks, morphological anomaly, restoration, crown fractures, and crown defects such as hypoplasia or hypocalcification. Post-bleaching teeth were excluded from this experiment. The teeth underwent a scaling operation using an Ultrasonic Scaler (Woodpecker UDS-A LED, China) to remove organic waste. They were then cleaned and disinfected with a 0.5% chlorine solution, and then stored in a physiological solution (Otsuka, Japan) at room temperature for 24 hours.

The tooth samples were prepared by trimming all roots using a low-speed handpiece (W&H, Bürmoos, Austria) to ensure a standardized distance of 7 mm from the cementoenamel junction to the base. All samples were fixed with self-cured acrylic (Hilon, England) into the silicone mold (25 mm × 25 mm × 13 mm), extending up to the cementoenamel junction, while ensuring that the long axis of the tooth remained in a vertical position. The buccal surface was polished with fluoride-free pumice paste for 15 seconds using a rubber polishing cup on the low-speed handpiece.

### Bleaching Protocol, Desensitizing Agent Application, and Bonding Bracket

The enamel surfaces of all specimens underwent bleaching using a 37% hydrogen peroxide solution (Pola Office + , SDI, Victoria, Australia) in accordance with the instructions provided by the manufacturer. A single bleaching cycle had a duration of 20 minutes and was repeated up to three times, resulting in a cumulative bleaching duration of 60 minutes. Subsequently, the samples underwent a rinsing process with distilled water, followed by desiccation using a dental chip blower. After bleaching, CPP-ACFP and CPP-ACP pastes were applied to the enamel surfaces in groups 2 and 3 and kept undisturbed for 4 minutes. The desensitizing agent was then removed by rinsing under flowing water. Then, all samples were bonded with a metal bracket (Pinnacle, Ortho Technology, West Columbia, South Carolina, United States), with 0.022-in slot, using Transbond Light Cure Adhesive Kit (Transbond XT Light Cure; 3 M Unitek, Monrovia, California, United States). Brackets were placed on the buccal surface, 4 mm from the cusp through the axis of the tooth. A force of 300 *g* was applied to each bracket, and any excess bonding resin was eliminated. After pressing the brackets on the buccal surface to produce uniform thickness, the brackets were then cured using a light-emitting diode curing unit (Woodpecker, Guilin, China) with a light intensity of 450 mW/cm ^2^ for 10 seconds from each side and then stored in a physiological solution (Otsuka Pharmaceutical Co., Ltd., Tokyo, Japan) at room temperature for 24 hours after bonding, prior to SBS analysis.

### SBS Assessment

The samples were placed in a universal testing machine (Pearson Panke Equipment Ltd., England), and the bracket–teeth contact was pushed with a force of 1 kN that moves equivalent to a crosshead speed of 1 mm/min by the chisel of the machine in the occlusogingival direction while moving downward until debonding. The maximum loads were measured in Newton (N) and subsequently converted into SBS, expressed in megapascals (MPa), by dividing it to the bracket base (9.14 mm ^2^ ).

### Adhesive Remnant Index Assessment

After the SBS assessment, the enamel surface was examined under a stereomicroscope (Olympus, Tokyo, Japan) at a magnification of ×10 to determine the residual adhesive after debonding. The scoring procedure was conducted on adhesive remnant index (ARI) with the following criteria 12 :

Score 1: All adhesive materials remained on the tooth
Score 2: > 90% of the adhesive materials remained on the tooth
Score 3: 10 to 90% of the adhesive materials remained on the tooth
Score 4: < 10% of the adhesive materials remained on the tooth
Score 5: No adhesive materials remained on the tooth

### Scanning Electron Microscopy and Energy-Dispersive X-ray Spectroscopy Evaluation

After SBS evaluation, the sample was analyzed using scanning electron microscopy (SEM) at ×10,000 magnification to ascertain the tooth surfaces. Then, an energy-dispersive X-ray spectroscopy (EDX) test was conducted to ascertain the calcium and phosphorous contents on the enamel surface after treatment. The operational parameters were 15 Kv of accelerating voltage, 10 nA of beam current, 30 to 45 seconds of counting time, and a working distance of 10 mm. The investigation was conducted using the field-emission SEM device model HITACHIS-4160 with a 5-nm resolution, 30-kV voltage, 500, 1,500, and 5,000 magnification, and 10 ^−10^ vacuum.

### Statistical Analysis

All statistical analysis was conducted using IBM SPSS Statistics version 25 (IBM Corp., Armonk, New York, United States). SBS data were subjected to a one-way analysis of variance (ANOVA) and Tukey's post hoc test, where ARI was analyzed using the Kruskal–Wallis and Mann–Whitney tests. The level of significance was set at *p* < 0.05.

## Results

Table 1 displays the descriptive statistics for the SBS values of all groups. The SBS data were normally distributed based on the Shapiro–Wilk test. Group 2 exhibited the highest mean SBS, which was recorded as 8.13 ± 0.91 MPa. In contrast, group 1 demonstrated the lowest mean SBS at 5.69 ± 0.91 MPa. ANOVA revealed significant differences in SBS data among the tested groups ( *p* < 0.05) ( Table 2 ). The Tukey post hoc test revealed that group 2 exhibited a statistically significant increase in its SBS compared with the other experimental groups. Furthermore, no statistical difference in SBS value was observed in the control groups 1 and 3 ( *p* > 0.05) ( Table 2 ).

**Table 1:** Descriptive statistics of SBS values (MPa)

Group	Number	Mean	Min	Max	Standard deviation	Standard error	95% Confidence for mean	
							Lower bound	Upper bound
1	9	5.6967	4.10	6.95	0.90746	0.30249	4.9991	6.3942
2	9	8.1289	5.56	9.91	1.32276	0.44092	7.1121	9.1456
3	9	6.8815	5.68	8.83	0.97118	0.32373	6.3071	7.5654

Abbreviations: CPP-ACFP, casein phosphopeptide-amorphous calcium fluoride phosphate; CPP-ACP, casein phosphopeptide-amorphous calcium phosphate; SBS, shear bond strength.

Note: Group 1 = control (without desensitizing agent).

Group 2 = fluor-based desensitizing agent (CPP-ACFP).

Group 3 = nonfluor-based desensitizing agent (CPP-ACP).

**Table 2:** Distribution score of ARI index

Group	Number	1	2	3	4	5
1	9	0	0	3	4	2
				(33.33%)	(44.44%)	(22.22%)
2	9	1	5	2	1	0
		(11.11%)	(55.56%)	(22.22%)	(11.11%)	
3	9	0	1	5	2	1
			(11.11%)	(55.56%)	(22.22%)	(11.11%)

Abbreviations: ARI, adhesive remnant index; CPP-ACFP, casein phosphopeptide-amorphous calcium fluoride phosphate; CPP-ACP, casein phosphopeptide-amorphous calcium phosphate.

Note: Group 1 = control (without desensitizing agent).

Group 2 = fluor-based desensitizing agent (CPP-ACFP).

Group 3 = nonfluor-based desensitizing agent (CPP-ACP).

**Fig. 1:**
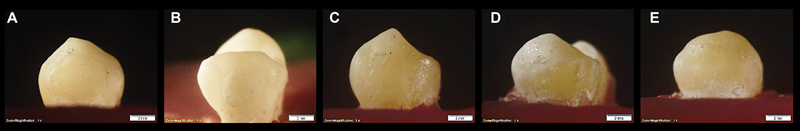
Adhesive remnant index under a stereomicroscope: ( **A** ) score 1, ( **B** ) score 2, ( **C** ) score 3, and ( **D** ) score 4, and ( **E** ) score 5.

Different ARI values under a stereomicroscope are shown in Fig. 1 . Table 3 presents the ARI of all the tested groups. The Kruskal–Wallis test revealed significant differences among the groups ( *p* < 0.05). In group 2, ARI was distributed between a score of 1 and 4, in which a score of 2 (55.56%) most frequently appears. In group 3, ARI was distributed in scores of 2 to 5, and the score of 3 most frequently appears (55.56%). Furthermore, in group 1 ARI was distributed between the scores of 3 and 5, and a score of 4 most frequently appears (44.44%). The Mann–Whitney test indicated significant differences in ARI between group 2 and other groups but no statistical difference was found between groups 2 and 3.

**Table 3:** The Mann–Whitney tests comparing the ARI index in all group tested

Group	Group 1	Group 2	Group 3
Group 1	–	0.003 a	0.279
Group 2	0.003 a	–	0.019 a
Group 3	0.279	0.019 a	–

Abbreviations: ARI, adhesive remnant index; CPP-ACFP, casein phosphopeptide-amorphous calcium fluoride phosphate; CPP-ACP, casein phosphopeptide-amorphous calcium phosphate.

Note: Group 1 = control (without desensitizing agent).

Group 2 = fluor-based desensitizing agent (CPP-ACFP).

Group 3 = nonfluor-based desensitizing agent (CPP-ACP).

a Significant differences between groups ( *p* < 0.05).

**Fig. 2:**
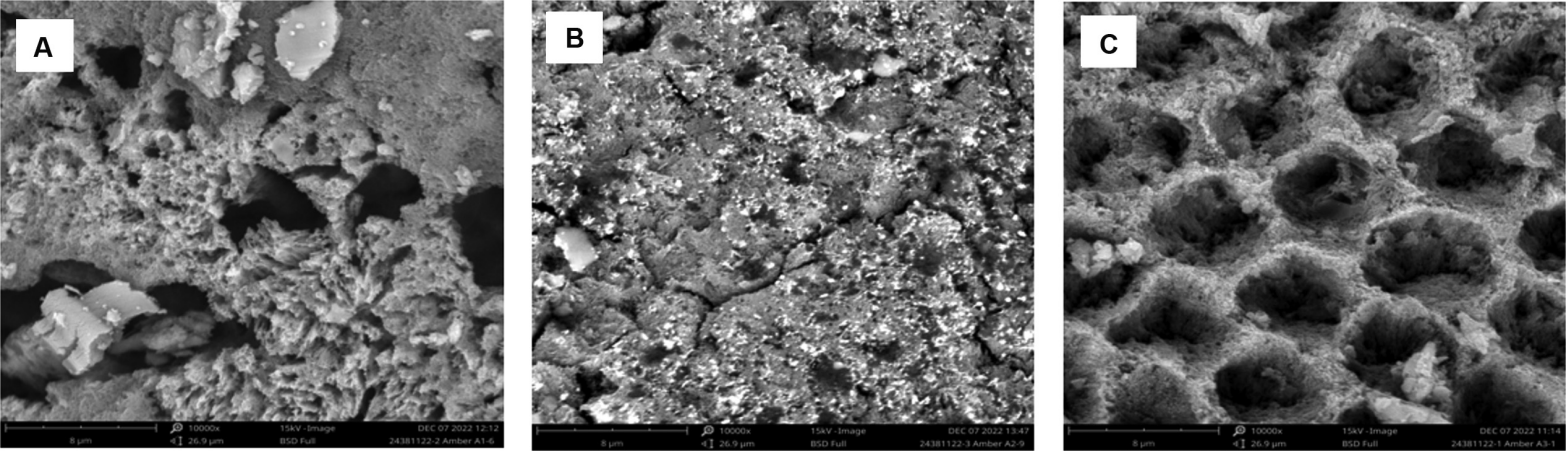
Scanning electron microscopy (SEM) images of the enamel surface after debonding bracket with magnification of ×10,000: ( **A** ) group 1 = control (without desensitizing agent), ( **B** ) group 2 = fluoride-based desensitizing agent (CPP-ACFP), and ( **C** ) group 3 = nonfluoride-based desensitizing agent (CPP-ACP). CPP-ACFP, casein phosphopeptide-amorphous calcium fluoride phosphate; CPP-ACP, casein phosphopeptide-amorphous calcium phosphate.

Fig. 2A shows an enamel surface with irregular porosity compared with Fig. 2B and C in which the sample was treated with desensitizing agents. Fig. 2B presents a sample with a fluoride-based desensitizing agent, and the enamel surface is covered with an even white layer so that the porosity is not too visible. Fig. 2C is an illustration of an enamel surface treated with a nonfluoride-based desensitizing agent, showing porosity with a white layer around the edges.

**Fig. 3:**
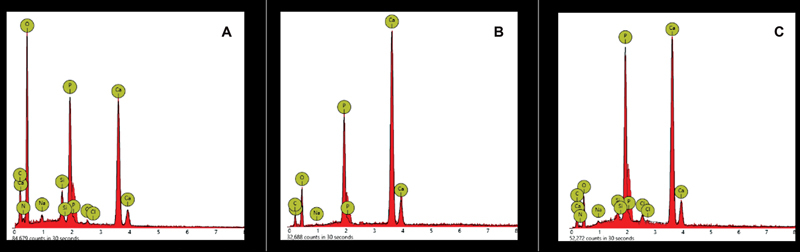
Elemental analysis by energy-dispersive X-ray spectroscopy of the enamel surface after the shear bond strength (SBS) test. ( **A** ) Group 1 = control (without desensitizing agent), ( **B** ) group 2 = fluoride-based desensitizing agent (CPP-ACFP), and ( **C** ) group 3 = nonfluoride-based desensitizing agent (CPP-ACP). CPP-ACFP, casein phosphopeptide-amorphous calcium fluoride phosphate; CPP-ACP, casein phosphopeptide-amorphous calcium phosphate.

Fig. 3A depicts the elemental analysis of the enamel surface after in-office bleaching therapy without the administration of desensitizing agents. Fig. 3B and C show the elemental analysis of the surface of the enamel after the administration of fluoride- and nonfluoride-based desensitizing agents following in-office bleaching, demonstrating the enamel surface with high values of calcium and phosphorus compared with the control group ( Fig. 3A ), as supported by the EDX graph.

## Discussion

This study assessed the effect of fluoride and nonfluoride-based desensitizing agents after in-office bleaching on the SBS of orthodontic metal brackets that bonded to the enamel surface with resin cementation. The results showed that the highest mean SBS was found in the CPP-ACFP desensitizing agent group (group 2), and the lowest mean SBS was noted in the control group (group 1). The findings suggest a potential beneficial impact of the desensitizing agent on the increasing SBS of the metal bracket. High or low concentrations of hydrogen peroxide in bleaching agents could cause demineralization, and this could be stopped by administering a desensitizing agent. 18 21 22 The basic mineral components of the mature enamel for remineralization include phosphate, hydroxyapatite, and calcium. When the pH is neutral and calcium and phosphorus ions are sufficient in the oral environment, the demineralization process can be stopped and converted to remineralization. 23 The EDX graph of treatment groups demonstrates the enamel surface with high values of calcium and phosphorus in comparison with the control group.

The results of SBS values revealed a significant difference between groups 3 and 2; however, no significant difference was found between groups 3 and group 1. This suggests that using a fluoride-based desensitizing agent after in-office bleaching will improve the SBS of metal brackets with resin cementation. The desensitizing agent acts as a remineralizing agent that forms a layer of calcium fluoride on the enamel surface. This layer removes residual oxygen so that it does not interfere with the attachment of the bracket to the enamel surface after in-office bleaching. 20 Remineralizing components such as fluoride, calcium, and ACP contained in desensitizing agents can minimize the side effects of bleaching treatments on the enamel thereby increasing the SBS of the brackets. 24 This finding also suggests that while the desensitizing agent aided in the remineralization of the enamel surface, the fluoride content was essential in enhancing the SBS of the metal bracket with resin cementation after in-office bleaching. Fluoride-based desensitizing agent (CPP-ACFP) contains fluoride that will create fluorapatite minerals, which are tougher and more acid-resistant than hydroxyapatite. 18 Bistey et al 22 stated that the remineralization process after bleaching can occur with the addition of low concentrations of fluoride. The addition of fluoride to CPP-ACP at low pH will increase the activity of hydroxy fluoride ion (HF0), allowing greater fluoride penetration into the lesion to accelerate remineralization. 25 A previous study reported that CPP-ACFP exerts a wider and faster remineralization effect than CPP-ACP. 26 Remineralization was delayed in group 3 because the absence of fluoride disrupted the metal bracket attachment; thus, the SBS test results did not differ significantly from the control group.

This investigation utilized a composite resin as an adhesive with a micromechanical retention mechanism on the acid-etched enamel surface. When phosphoric acid is applied to the enamel surface, hydroxyapatite crystals are dissolved to produce microporosity. Microporosity is filled with liquid monomer during polymerization, resulting in the formation of a micromechanical bond between the resin and enamel. 27 The enamel surface undergoes morphological changes and a decrease in microhardness during in-office bleaching, which may make the pores more irregular because of the etching acid used, disrupting the bracket attachment if remineralization is not performed. Kutuk et al 24 stated that the decrease in microhardness after bleaching could be corrected by administering a desensitizing agent containing fluoride and/or calcium.

Free radical residues that are released by hydrogen peroxide cause the inhibition of polymerization and the infiltration of resin-based materials onto the enamel surface after in-office bleaching. 28 The negative effects of free radical residues can be alleviated by adding low amounts of fluoride after bleaching. The calcium fluoride layer will eliminate residual oxygen so that it does not interfere with the bracket's attachment to the enamel surface after in-office bleaching. 20 The penetration of fluoride into the lesion will accelerate the remineralization process and induce wider and faster remineralization than using a nonfluoride-based desensitizing agent. 25 26 Wider and faster remineralization will minimize side effects after bleaching on the tooth surface structure, so that the polymerization process during the bonding bracket must not be disrupted. The polymerization process of the adhesive was not disrupted, causing the SBS value to be higher than that in group 3.

Analysis of ARI data revealed a statistically significant difference between groups 3 and 2; however, no significant difference was found between groups 3 and 1. The ARI distribution in group 3 revealed that the highest number of samples scored 3. When compared with the ARI distribution in group 2, this distribution demonstrates that little adhesive remained on the tooth surface of more samples after the SBS test. The fluoride in the desensitizing agent is important in the remineralization of the enamel surface after in-office bleaching. The addition of flour to CPP-ACP forms CPP-ACFP, which exhibits a notable increase in microhardness and reduces enamel demineralization. 29 SEM images in groups 1 and 3 were similar, in that porosity was still visible after the SBS test; however, porosity was not as visible in group 2. This condition occurred because more adhesive materials were left on the tooth surface in group 2 so that tooth porosity was covered, whereas in groups 3 and 1, not much adhesive materials were left on the tooth surface, so tooth porosity was visible by SEM observation. The lack of interference with bracket attachment was indicated by high SBS values and many adhesive materials were left on the tooth surface after the SBS test in group 2 compared with group 3. The results of this study are in line with the findings of Henkin et al 30 who stated that large amounts of residual adhesive material on the enamel surface were associated with higher SBS values.

The SBS value in group 2 was 8.13 MPa, which was lower than the maximum clinically acceptable bracket attachment strength limit but greater than the ideal range of forces to withstand masticatory and orthodontic forces. According to Reynolds, 31 a resistance ranging from 5.9 to 7.8 MPa is adequate to sustain masticatory pressures and can tolerate orthodontic forces that are applied to the brackets. Although SBS values were higher than the ideal force, this was not a problem because according to Retief, 32 enamel fractures will occur if the force reaches ≥ 13.5 MPa.

The results of this study indicated that administering a CPP-ACFP after in-office bleaching increases the SBS of metal brackets while remaining below the maximum force that might induce enamel fracture. The administration of a CPP-ACFP minimizes demineralization after in-office bleaching and promotes faster remineralization, ensuring that the bracket's adhesion is not disrupted, as seen by the large amounts of adhesive materials remaining on the tooth surface after the SBS test. Based on the findings of this study, patients can undergo in-office bleaching and bonding of metal brackets within the same visit.

## Conclusion

Within the limitations of the study, the application of CPP-ACFP desensitizing agent after in-office bleaching was effective in increasing the SBS of metal brackets with resin cementation. However, additional clinical and laboratory studies are needed to confirm the efficacy and potency of CPP-ACFP desensitizing agent with different time intervals between in-office bleaching and bonding bracket.
